# Research on structural parameter optimization of a new axial inlet hydrocyclone separator based on response surface optimization method

**DOI:** 10.1371/journal.pone.0295978

**Published:** 2024-01-02

**Authors:** Yong Zhang, Yan Zhang, Wei Liu, Hongguang Yi, He Wang

**Affiliations:** 1 College of Intelligent Manufacture, Taizhou University, Taizhou, Zhejiang, China; 2 College of Civil and Architectural Engineering, Taizhou University, Taizhou, Zhejiang, China; 3 Collegel of Mechanics Science and Engineering, Northeast Petroleum University, Daqing, Heilongjiang, China; Sivas Cumhuriyet University, TURKEY

## Abstract

Aiming at the problem that the current hydrocyclone separator is affected by multiple structural parameters and there is interaction between the multiple structural parameters, it is difficult to determine the optimal structure. Taking the new axial inlet hydrocyclone separator as the research object, a fast parametric optimization method based on response surface optimization method is proposed. The overflow outlet diameter, overflow tube depth and small cone length, which have significant influence on the separation efficiency of the axial inlet hydrocyclone separator, are selected as the optimal variables, and the cyclone separation efficiency is selected as the response index. A mathematical driving model between the response index and the optimal variables is constructed by using the second-order polynomial basis function. The optimal structural parameters of the new axial inlet hydrocyclone separator are obtained through the response optimization of the parameter variables in the global response range through the mathematical driving model, and the numerical simulation method and laboratory test are double verified. The results demonstrated that the axial inlet hydrocyclone achieved the highest separation efficiency within the studied operational parameter range when the overflow pipe diameter was 6mm, the overflow pipe depth was 20mm, and the small cone length was 60mm. The separation efficiency improved from 89% to 93%. The rapid optimization of the structural parameters of the axial inlet hydrocyclone was successfully accomplished using the response surface optimization method.

## Introduction

As the main oil fields in China enter the development period of high water cut, the traditional surface oil-water separation technology will lead to the increasing cost of crude oil exploitation and is not suitable for the economic requirements of effective exploitation [1, 2]. At this time, the technology of downhole oil-water separation and reinjection in the same well can effectively solve the problems of high treatment cost and low mining efficiency of high water cut lifting to the surface in the Wells of waterflood oilfield [3–5]. However, the core of the same well injection-production technology is the downhole oil-water separation technology, and the downhole oil-water separation is mainly based on cyclone separation, and the cyclone separation device includes hydraulic cyclone separator. The structure of the hydrocyclone separator is very important to the separation performance [6]. For example, changes in structural parameters such as the diameter of the overflow outlet, the length of the overflow tube, and the length of the cone can seriously affect the separation efficiency of the hydrocyclone separator. At present, the single factor control variable optimization method is often used to determine the optimal structural parameters of hydraulic cyclone separators. During the single factor control variable optimization process, only the optimal parameters of a single factor can be determined, and the interaction between multiple factors cannot be considered. The obtained optimal parameters have certain limitations, and the optimization process is also more complex and time-consuming [7–9]. Therefore, it is of great significance to find a fast optimization method suitable for the influence of multiple structural parameters of hydrocyclone to guide the design and selection of hydrocyclone, improve the separation performance of hydrocyclone, and accelerate the further promotion and application of injection production technology in the same well [10].

In recent years, many scholars have carried out research on the optimization of structural parameters of hydrocyclone separators, including numerical simulation and laboratory experiment.

Liu [11] concluded through research that the overflow port diameter of the hydrocyclone separator would affect the separation efficiency of the hydrocyclone. Wei [12] found through research that the cone angles of different cone sections had an impact on the flow field performance and separation performance of a hydrocyclone separator, while the small cone section mainly affected the separation ratio by affecting the axial velocity. Liu [13] experimentally studied the diameter of the overflow port under different openings by setting baffles on the overflow pipe, which was equivalent to adjusting different overflow pipe diameters. It was found that the change in the diameter of the overflow pipe in the hydrocyclone separator can affect the separation efficiency of the hydrocyclone separator. Zhao [14] studied the effect of overflow tube extension length and overflow tube diameter on a porous overflow tube type hydrocyclone separator. Jiang [15] conducted simulation results analysis on the inlet structure of a new type of axial dehydration hydrocyclone. Gao [16] used the principle and method of Computational fluid dynamics to conduct numerical simulation of the internal flow field of the hydrocyclone at multiple levels of the bottom flow port diameter and cone angle to reveal the influence of two factors on the flow field of the hydrocyclone. The above literature mainly adopts the single-factor optimization study of the control variable method, ignoring the interaction of multiple factors on the separation performance of the hydrocyclone separator, and the obtained optimization results cannot be guaranteed to be the optimal results within the global response range. Therefore, this article proposes an optimization method based on response surface method to optimize the structure parameters of the hydrocyclone separator under the influence of multi-factor interaction, so as to realize the rapid optimization of the hydrocyclone separator.

## Model size and parameter setting

### Model establishment

**Fig 1:**
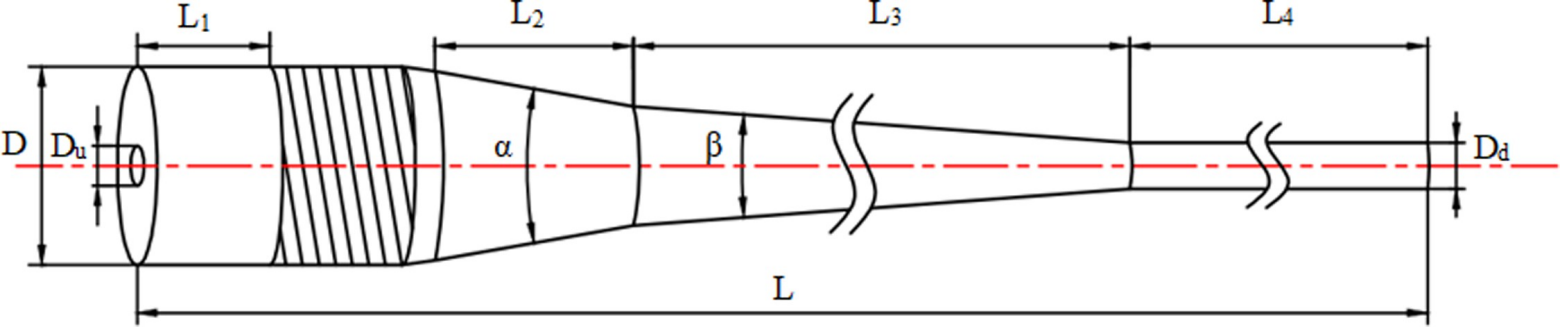
Fluid domain model of a new axial inlet hydrocyclone separator.

Parametric modeling is carried out according to the actual size of the hydrocyclone. The initial Geometric modeling is shown in Fig 1, and the name and size of the initial geometric structure parameters are shown in Table 1. The model adopts an axial inlet design, with a conical drainage section set up inside the inlet. The liquid from the axial inlet enters the spiral flow channel after being drained by the conical body for acceleration. The accelerating liquid forms a continuous vortex to drive the liquid to rotate and move towards the conical section. A low pressure zone near the axis is formed and a high pressure zone near the side wall is formed. The final dense oil phase is discharged through the upper overflow pipe, and the denser water phase is discharged through the bottom flow pipe to achieve the final oil-water separation [17].

**Table 1:** Initial model size of an axial hydrocyclone separator.

Parameter	Symbol	Size/mm	Parameter	Symbol	Size/mm
**Diameter of inlet**	D	60	**Diameter of overflow outlet**	Du	10
**Depth of overflow tube**	H	15	**Length of inlet section**	L_1_	60
**Length of middle cone**	L_2_	80	**Length of small cone**	L_3_	535
**Length of underflow**	L_4_	500	**Length of overall**	L	1245
**Angle of big cone**	α	20°	**Angle of small cone**	β	5°

## Finite element meshing

**Fig 2:**
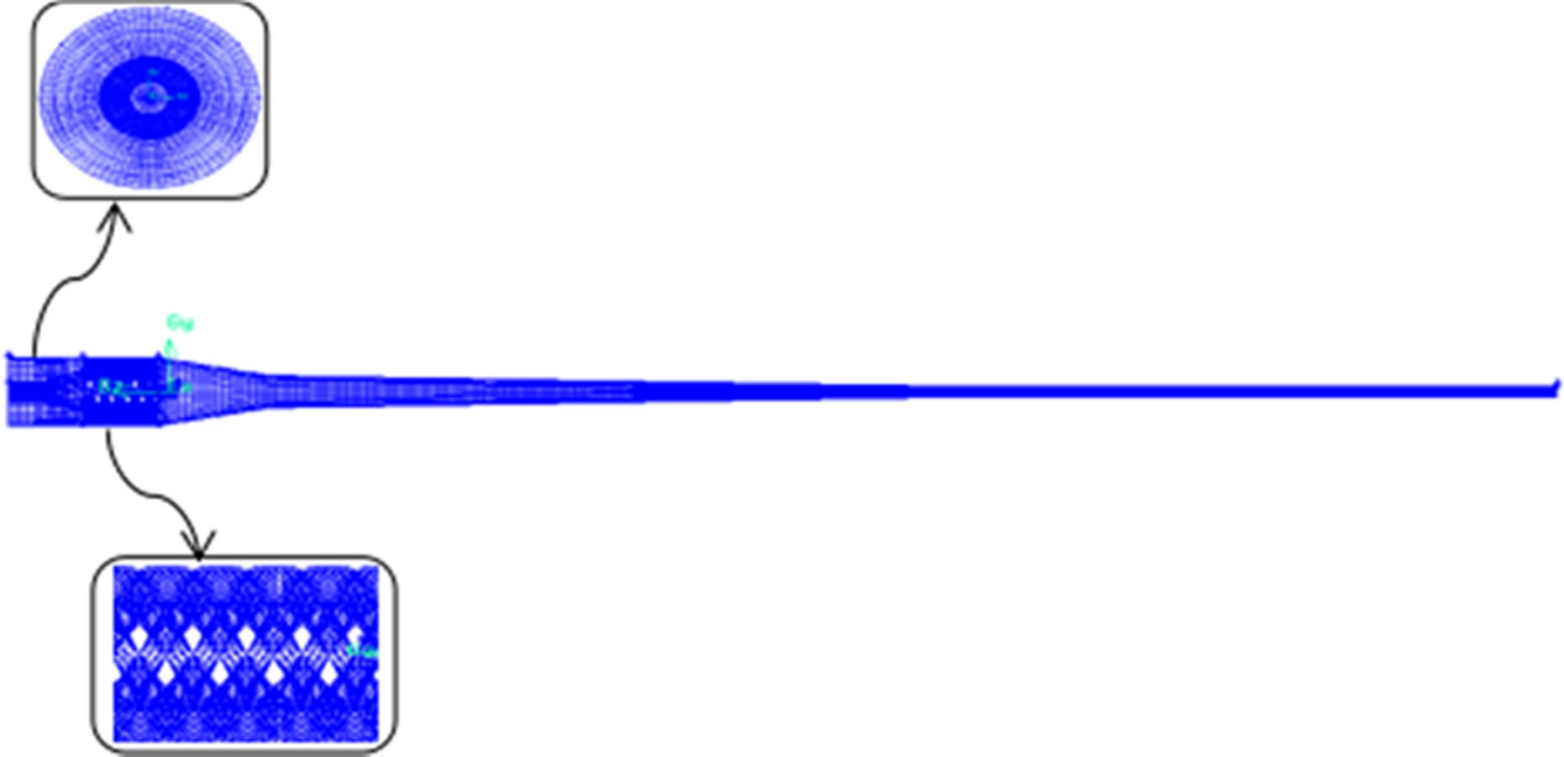
Finite element meshing of fluid models.

Because the hexahedron grid is easy to realize the orthogonality principle at the wall surface, the calculation accuracy is high and the speed is fast, which is very suitable for the simulation calculation of multiphase flow, so the hexahedron grid is divided for the hydrocyclone separator [18]. To test the independence of the grid, four different types of unit grids were divided: 258452 (separation efficiency of 84.1%), 296342 (separation efficiency of 84.6%), 349815 (separation efficiency of 87.1%), and 378539 (separation efficiency of 87.5%). As the simulation values of the latter two separation efficiencies are similar, in order to improve the calculation speed, a mesh division model with a finite cell grid number of 349,815 and a node number of 88453 is selected, in which the mesh efficiency of the finite element model is 99.8%, as shown in Fig 2.

## Parameter setting

The simulated medium is oil-water two-phase liquid. Among them, the water phase is a continuous phase medium and the oil is a discrete phase medium. The density of the water phase is 998.2kg /m^3^. The viscosity of the water is 1.003×10^-3^Pa·s. The density of the oil phase is 889kg /m^3^. The viscosity of the oil is 1.006Pa·s, and the volume fraction of the oil is 3%. In order to match the temperature with the indoor experimental temperature, the liquid temperature in the hydrocyclone is set to 298k. Boundary types of inlet and outlet are velocity inlet and outflow respectively. For the remaining parameter settings are shown in Table 2. Mixture model was adopted as the multiphase flow model. Steady solution of implicit solver with pressure datum algorithm is selected, first order upwind scheme is used as a convection-diffusion term, SIMPLEC pressure-velocity coupling algorithm is selected. Study showed that the turbulence model in a rotating flow field has the highest effect on numerical simulation results, reaching about 15% [19]. About the advantages and disadvantages of various turbulence models in the rotating flow field, Wang et al. [20] have done a more comprehensive discussion. At present, the Reynolds Stress Model has been widely recognized and applied in simulation of continuous phase flow field [21], and it has gradually become a basic tool for engineering turbulence calculation [22]. Based on research experience, the Reynolds Stress Model completely renounces the hypothesis of eddy viscosity, which is in line with the strong swirling turbulence in hydrocyclones, so it is used for turbulent calculation model [23–26].

**Table 2:** Operation parameter settings.

Fluid velocity/ (m/s)	Overflow split ratio	Underflow split ratio	turbulence intensity
1.2	0.2	0.8	5%

The wall function method is used for the near-wall region with low Reynolds number. The wall function rule no longer solves the governing equation for the viscous bottom layer and the transition layer, but uses the empirical formula to connect the flow field parameters of the near-wall surface with the corresponding values on the wall, and connects the physical quantities of the viscous influence region with the variables formed by the turbulence model in the turbulent core region. The wall function rule can be described by the following equation:

$$u^{+}=\frac{u}{u_{t}}$$ (1)

$$u_{\tau }=\sqrt{\frac{\tau_{w}}{\rho }}$$ (2)

$$\tau_{w}=\frac{\rho C_{\mu }^{1/4}k^{1/2}u_{f}}{u^{+}}$$ (3)

$$y^{+}=\frac{\Delta y\rho u_{\tau }}{\mu }=\frac{\Delta y\rho u_{f}}{\mu u^{+}}$$ (4)

Where ***u***^**+**^ and ***y***^**+**^ represent dimensionless parameters of velocity and distance along the normal side of the wall, respectively; **Δ*y*** and ***y***^**+**^ represent the distance to the normal of the wall; ***u***_***τ***_ is the friction velocity of the wall; ***τ***_***w***_ is the wall shear stress.

Some results show that the boundary layer flow field structure changes according to the change of ***y***^**+**^ range [27]. When ***y***^**+**^ is greater than 11.63, the fluid flow is turbulent, and when ***y***^**+**^ is less than 11.63, the fluid flow is laminar. Therefore, the calculation formula using the standard wall function is as follows:

$$y^{+}\;<\;11.63\;\;u^{+}=y^{+}$$ (5)

$$11.63\;<y^{+}\;<\;300\;\;u^{+}=\frac{1}{k}\;\text{ln}y^{+}+B=\frac{1}{k}\text{ln}(Ey^{+})$$ (6)

Where ***k*** is the Feng·Karman constant; ***B*** and ***E*** are constants related to surface roughness. For the inner wall of the cyclone, ***k*** = 4, ***B*** = 5.5, ***E*** = 9.793 are usually taken.

When the wall function method is used to deal with the wall problem, it is necessary to correctly deal with the position of the grid points in the boundary layer, and the location of the first grid point is particularly important. The usual choice is that when ***y***^**+**^ is greater than 30 and ***y***^**+**^ is less than 300, and calculated as:

$$y^{+}=\frac{\Delta y_{p}\rho u_{y}}{\mu u^{+}}$$ (7)

Where **Δ*y***_***p***_ is the distance between the first node of the grid and the wall; ***u***_***y***_ is the mean time velocity of the fluid at point y.

## Optimal design of response surface

Compared with the orthogonal experimental design method, the optimal scheme obtained by the response surface method can only be limited to the given level, rather than the optimal scheme within the experimental range, and the optimal combination of design variables and the optimal value of the response target can not be obtained. In addition, in many cases, the interaction between some factors cannot be ignored, otherwise the optimization results are not reliable, so a large number of experiments are still needed to judge the second-order, third-order and even higher-order interactions. However, the response surface method can continuously carry out optimization analysis on the levels of multiple impact factors in the optimization process, which can overcome the defects that the orthogonal design can only optimize the design analysis of each isolated point, and has the advantages of fewer test times, good prediction performance, and considering the interaction between factors. So it is more suitable for the parameter optimization design of the hydraulic cyclone separator.

### Response surface theory

The response surface method based on experimental design is to design a finite number of test schemes for the set of sample points in the specified design space, and then replace the real response surface with an infinite approximation scheme to fit the global response of the system according to the test design results. Therefore, the key to construct the response surface model is to clarify the relationship between the design variable and the analysis target, and select a suitable functional form to describe the relationship between the current design variable and the analysis target [28, 29]. The methods of constructing response surfaces include fitting polynomial, exponential function and logarithmic function, as well as approximation methods such as neural network, among which polynomial approximation model is widely used.

The functional expression between the system response Y after design of experiments and the design variable *x* can be expressed as [30, 31]:

$$Y=\tilde{y}(x)+\delta$$ (8)

Where $\tilde{y}(x)$ represents the approximate function of the unknown function, ***δ*** represents total error.

Among them, if the Quadratic Response Surface Test Box-Behnken Design (BBD) and Central Composite Design (CCD) design methods are used to approximate the relationship between the system design variables and response indicators, a second-order calculation model is required to approximate the response surface.


$$\tilde{y}(x)=\beta_{0}+\sum_{i=1}^{k}\beta_{i}\chi_{i}+\sum_{i=1}^{k}\beta_{i}\chi_{i}{}^{2}+\sum_{i=1}^{k}\beta_{ij}\chi_{i}\chi_{j}+\varepsilon$$ (9)

Where $\beta_{i}、\beta_{ii}、\beta_{ij}$ represents odd function, $\chi_{i}、\chi_{j}$ represents basis function.

### Response surface test design

There are a variety of response surface test design methods [32], among which Box-Behnken Design (BBD) and Central Composite Design (CCD) are frequently used in response surface test design, which provides a better understanding of the process than the standard experimental methods, because it is able to predict how the inputs influence the outputs in a complex process where different factors can interact among themselves [33, 34]. This article adopts the star point combination design method of CCD, and takes the diameter of the overflow, the insertion depth of the overflow tube, and the length of the small cone as the design factors that have the greatest impact on the structure of the axial guide cone hydrocyclone (represented by *x*_1_、*x*_2_ and *x*_3_ respectively). The oil-water separation efficiency of the axial hydraulic cyclone separator is evaluated as the evaluation index, and response surface optimization design experiments are conducted. Central Composite Face (CCF) design belongs to the face center combination design method in CCD design, which consists of adding axial points and center points on the basis of the cubic points of the two level factorial design. The central composite face-centered design (CCF) is chosen as the optimal design for hydrocyclone due to its advantages in optimizing multi-factor problems and optimizing the number of experiments [6]. The CCF method takes 3 levels for each factor and encodes them with (0, ±1) as the center point. The initial structural model size value is the optimal value point of each single factor after the orthogonal Design of experiments has been used. Therefore, the initial structural size value is the central point value of the CCF face center combination design, and the ±1 structural design limit value is the lowest and highest level value. See Table 3 for details. The specific factor levels are shown in Table 3.

**Table 3:** Design factors and levels.

Options	Code symbol	Level		
		-1	0	1
**Diameter of overflow outlet/mm**	*x*_1_	6	10	14
**Depth of overflow tube /mm**	*x*_2_	10	15	20
**Length of small cone/mm**	*x*_3_	60	80	100

After each factor and level value are input into the test design software in sequence, 19 groups of test data are generated, and the computational fluid dynamics numerical simulation software is used to simulate the separation efficiency results for each group of matched design variables. The calculation formula of separation efficiency is shown in formula (3), and the final results of the response surface design scheme are shown in Table 4.


$$E_{j}=1-\frac{C_{d}}{C_{i}}$$ (10)

Where E_j_ represents the separation efficiency of hydrocyclone separator, C_d_ represents the oil concentration at bottom flow outlet, C_i_ represents the oil concentration at the inlet.

**Table 4:** Response surface design scheme and result.

Number	Diameter of overflow outlet/mm	Depth of overflow tube /mm	Length of small cone/mm	Separation efficiency
1	14	10	60	86.96
2	10	20	80	90.28
3	10	15	80	89.22
4	6	15	80	89.87
5	6	20	100	91.69
6	14	10	100	90.04
7	10	15	80	89.22
8	10	15	80	89.22
9	10	15	80	89.22
10	10	15	100	91
11	14	20	60	90.8
12	6	20	60	92.58
13	14	15	80	88.95
14	6	10	60	89.19
15	10	15	60	89.38
16	6	10	100	91.16
17	14	20	100	91.21
18	10	10	80	89.23
19	10	15	80	89.22

### Response model and test

Using the second-order (Quadratic) model settings in Design Expert software, the constant term, first-order term, quadratic term (interaction term), square term (surface action term), and significance tests that affect the quadratic model in the analysis of variance are shown in Table 5.

**Table 5:** Analysis of variance of experimental results.

Source	Sum of squares	Degrees of freedom	Mean square	F value	P value	Remarks
**Model**	28.11	9	3.12	33.63	<0.0001	Highly significant^a^
*x*_1_	4.26	1	4.26	45.91	<0.0001	Highly significant^a^
*x*_2_	9.96	1	9.96	107.24	<0.0001	Highly significant^a^
*x*_3_	3.83	1	3.83	41.25	0.0001	Highly significant^a^
*x*_1_ *x*_2_	0.1485	1	0.1485	1.60	0.2378	Not significant^c^
*x*_1_ *x*_3_	0.7260	1	0.7260	7.82	0.0209	Significant^b^
*x*_2_ *x*_3_	3.82	1	3.82	41.16	0.0001	Highly significant^a^
** $x_{1}{}^{2}$ **	0.001	1	0.001	0.0104	0.9210	Not significant^c^
** $x_{2}{}^{2}$ **	0.3617	1	0.3617	3.89	0.0799	Not significant^c^
** $x_{3}{}^{2}$ **	1.74	1	1.74	18.77	0.0019	Highly significant^a^
**Residual**	0.8359	9	0.0929			
**Total deviation**	28.95	18				

^a^Highly siginificant means that the P value is less than 0.01.

^b^Siginificant means that the P value is less than 0.05.

^c^Not siginificant means that the P value is greater than 0.05.

From Table 5, it can be seen that the significance of the correlation coefficient is represented by large F values and small P values. According to Table 5, the F value of the model is equal to 33.63 and the P value is less than 0.0001, indicating that the model is highly significant and has high fitting accuracy. Therefore, this approximate model can be chosen for later optimization design. At the same time, it can be seen that the three design factors selected as overflow tube diameter, overflow tube insertion depth and small cone section length are highly significant. Among them, the interaction between flow tube diameter and overflow tube insertion depth is not significant, while the interaction between flow tube diameter and small cone section length is significant, and the interaction between overflow tube insertion depth and small cone section length is significant.

Because the influence of various factors on the separation efficiency of a hydrocyclone is nonlinear, this article conducts multiple regression fitting based on the separation efficiency test data in Table 4, and obtains the regression mathematical driving model for the separation efficiency as follows:

$$\begin{array}{c} Separationefficiency=97.37-0.59x_{1}+0.25x_{2}-0.22x_{3}\\ +0.0068x_{1}x_{2}+0.0038x_{1}x_{3}-0.0069x_{2}x_{3}\;+0.0012x_{1}^{2}+0.015x_{2}^{2}+0.0020x_{3}^{2} \end{array}$$

The results of error statistical analysis on the polynomial regression model of hydrocyclone separation efficiency are shown in Table 6. R^2^ represents the correlation coefficient of the multiple regression equation, and the larger the R^2^ value and the closer it is to 1, the better the correlation. Adjust R^2^ represents the corrected correlation coefficient, and Predict R^2^ represents the predicted correlation coefficient. If the value of Adjust R^2^ is highly close to the value of Predict R^2^ and satisfies (Adjust R^2^-Predict R^2^)<0.2, it indicates that the regression model can fully demonstrate the engineering problem being studied. If the difference percentage C.V.% is less than 10%, it indicates that the credibility and accuracy of the model obtained from the experiment are more accurate. Adeq Precision is the precision of the regression model. If the value is greater than 4, it indicates that the model is reasonable. From Table 6, it can be seen that the correlation coefficient R^2^ = 0.9711 of the regression model for the separation efficiency of the hydrocyclone separator approaches 1, indicating a good correlation of the regression model for the separation efficiency of the hydrocyclone separator. Adjust R^2^-Predict R^2^ = 0.16<0.2, indicating that the regression model can fully demonstrate the engineering problem of the separation efficiency of the studied hydrocyclone separator. CV% = 0.34%<10%, indicating that the model obtained from the experiment is reliable and has high accuracy. Adeq Precision = 23.91>4, indicating that the model is reasonable. Through model testing of the separation efficiency of the hydrocyclone separator, it was found that the regression model conforms to the above testing principles and has good adaptability.

**Table 6:** Error statistics of regression models.

Item	Value	Item	Value
Std Deviation	0.3048	R^2^	0.9711
Mean	89.92	Adjust R^2^	0.9423
C.V.%	0.3389	Predict R^2^	0.7822
PRESS	861.65	Adeq Precision	23.9129

**Fig 3:**
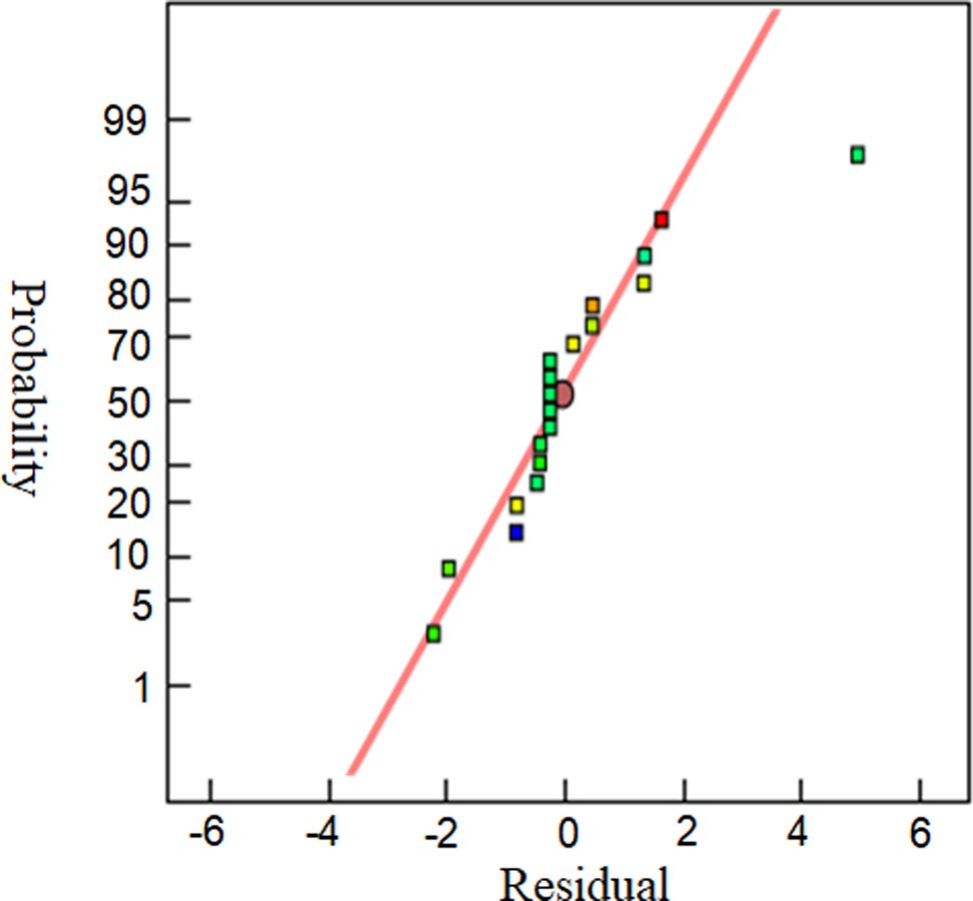
Probability plot of residual normal distribution.

**Fig 4:**
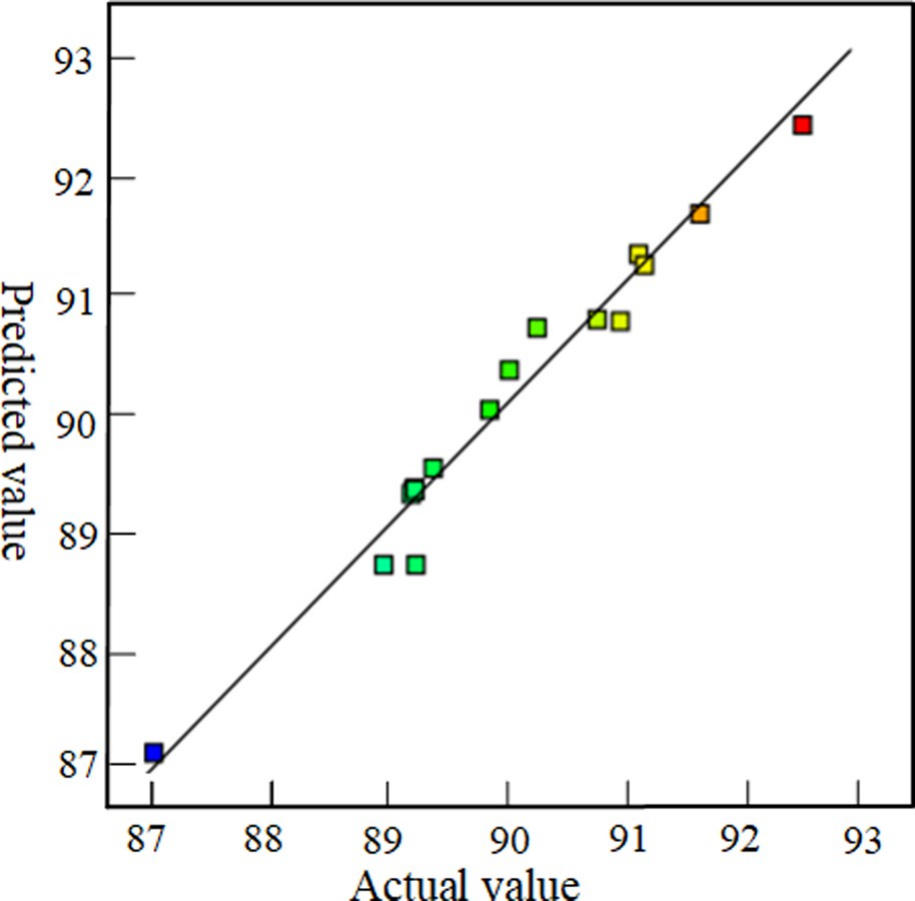
Distribution map of actual and predicted values.

The residual probability curve of the mathematical driving equation for response surface optimization is shown in Fig 3, and the actual and predicted values are shown in Fig 4. It can be seen from the residual probability curve and the curve between the actual value and the predicted value that the distribution points are scattered around the fitting line, indicating the accuracy of the mathematical driven model prediction.

### Response surface and contour plot

According to the P value in Table 5, the interaction between the overflow outlet diameter and the overflow tube insertion depth is not significant, but the interaction between the overflow outlet diameter and the length of the small cone section, the overflow tube insertion depth and the length of the small cone section is significant. Based on the three-dimensional response surface diagram and contour diagram of the interaction between the test factors obtained from the response surface optimization results, the effects of the interaction between the overflow outlet diameter and the length of the small cone section and the overflow tube insertion depth and the length of the small cone section on the separation efficiency of the axial hydrocyclone separator were analyzed respectively.

**Fig 5:**
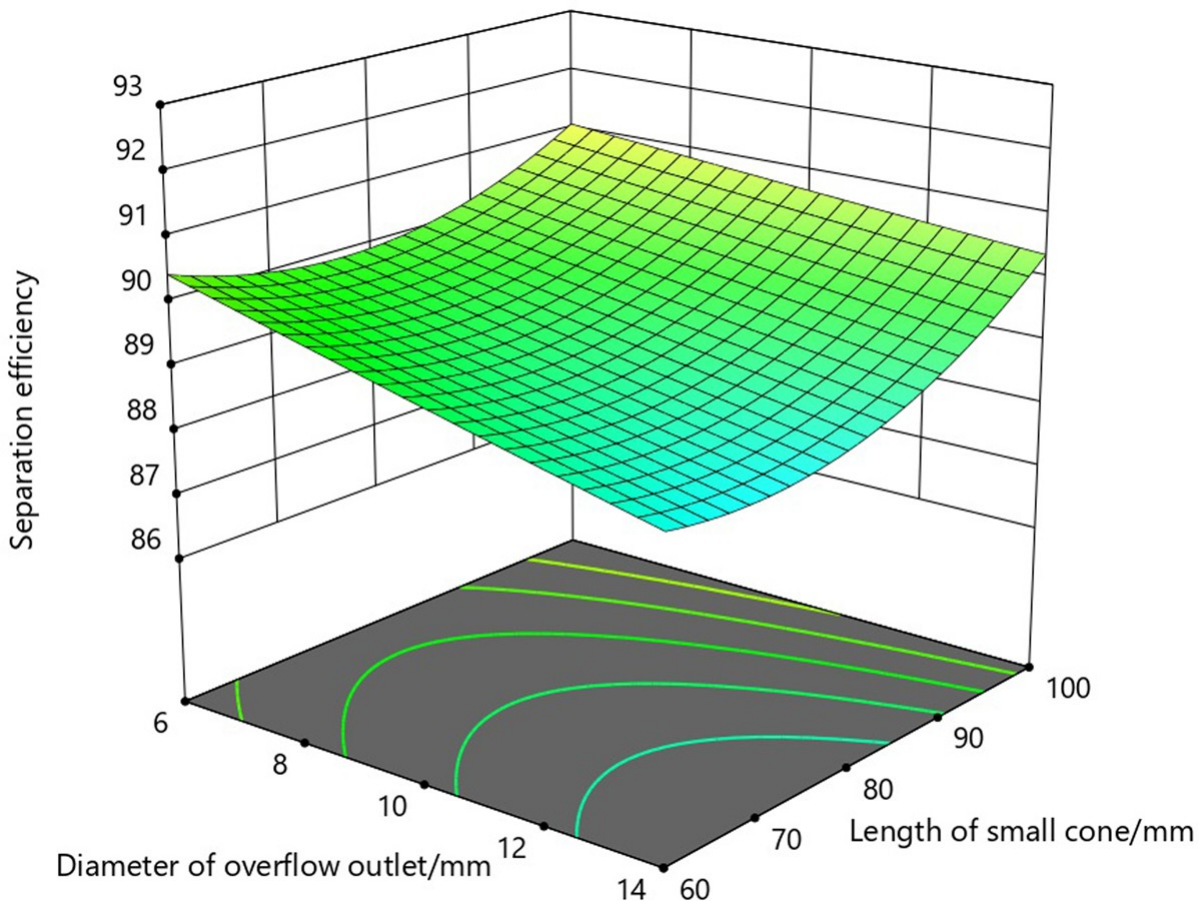
Three-dimensional response surface diagram of overflow outlet diameter and small cone length.

**Fig 6:**
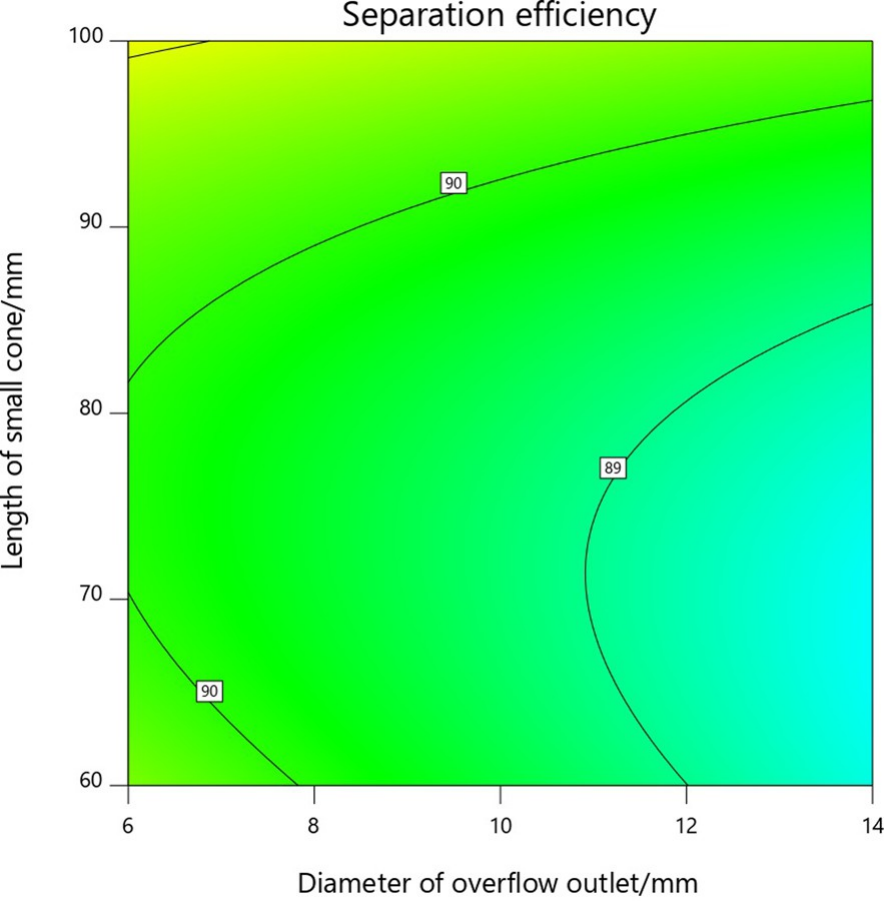
Contour diagram of overflow outlet diameter and small cone length.

As can be seen from the three-dimensional response surface diagram in Fig 5 and the contour diagram in Fig 6, the separation efficiency of the hydrocyclone separator has a maximum value in the region when the optimum diameter of the overflow tube is 6-8mm and the length of the small cone section is 60-70mm.

**Fig 7:**
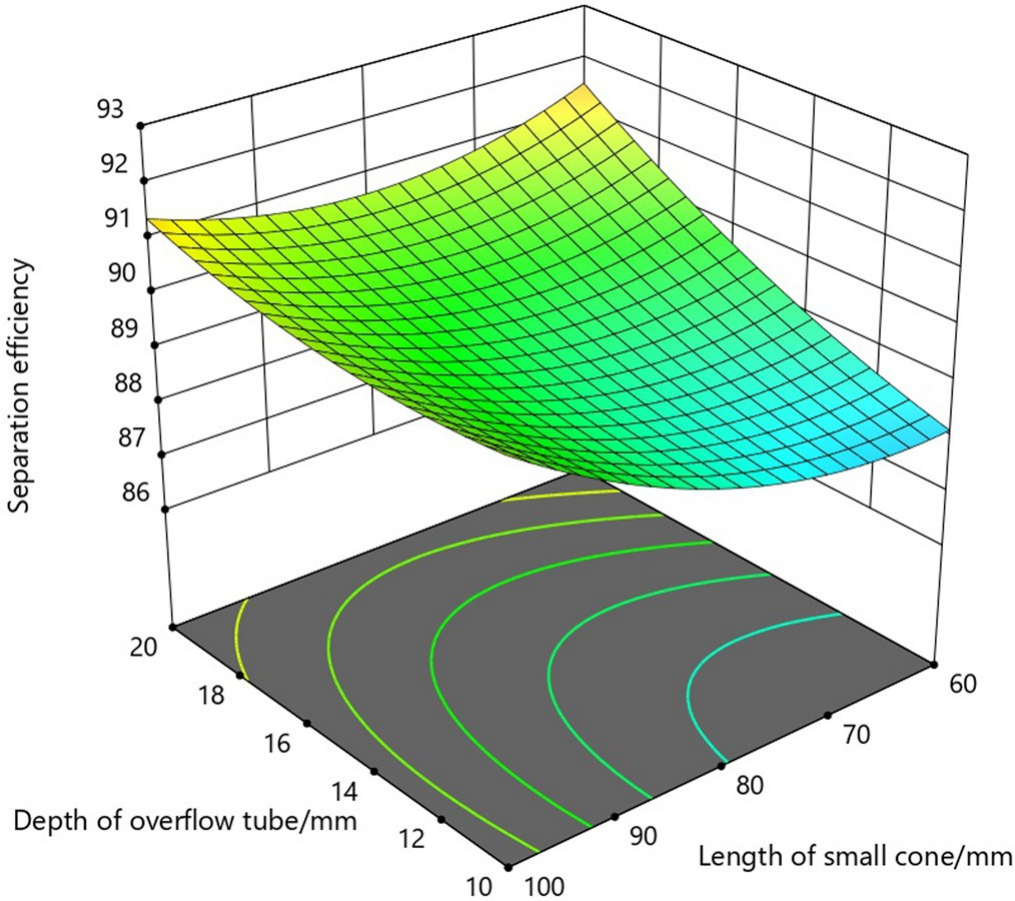
Three-dimensional response surface diagram of overflow tube insertion depth and small cone length.

**Fig 8:**
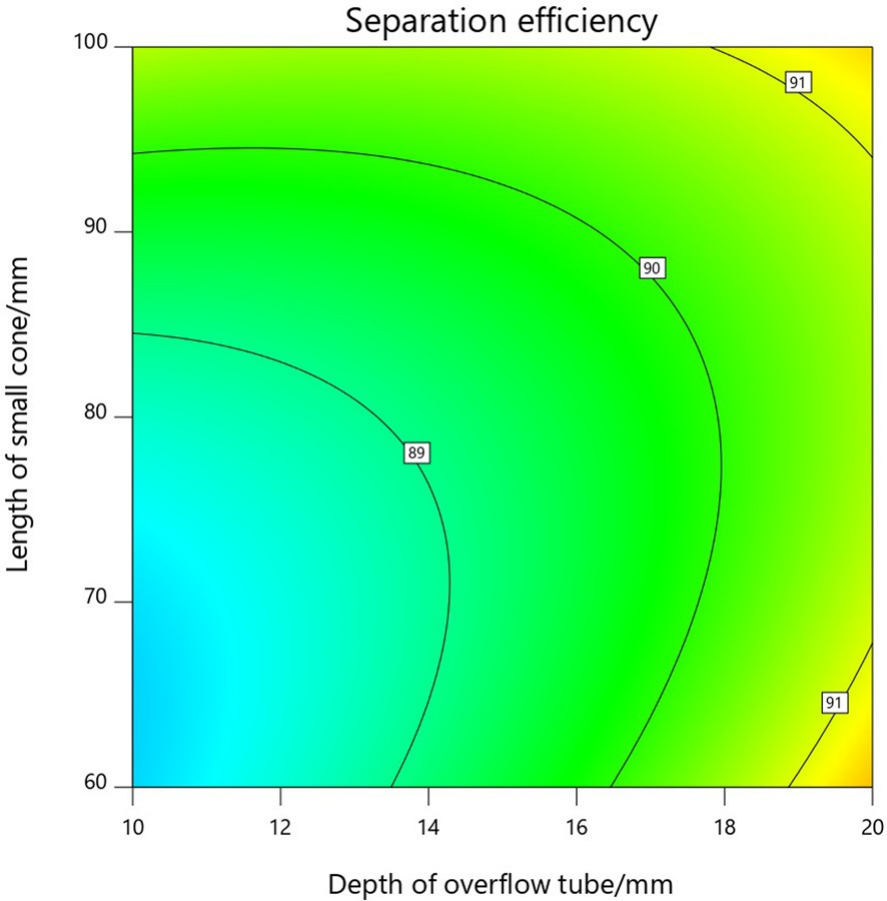
Contour diagram of overflow tube insertion depth and small cone length.

As can be seen from the three-dimensional response surface diagram in Fig 7 and the contour diagram in Fig 8, the optimal range of the overflow tube insertion depth is 16-20mm, and the separation efficiency of the hydrocyclone separator has a maximum value in the region when the length of the small cone section is 60-70mm.

According to the results of response surface cloud image analysis, it is found that the hydrocyclone separator has the optimal separation efficiency in the global response range under the interaction of design factors. Based on the mathematical driving model, it is determined that when the overflow outlet diameter is 6mm, the overflow tube insertion depth is 20mm and the cone section length is 60mm, the structure size of the hydrocyclone separator is the optimal structure size, and the oil-water separation efficiency of the hydrocyclone separator can reach the maximum value in the global response range. The data-driven model predicted the separation efficiency of the hydrocyclone separator before and after optimization, as shown in Table 7.

**Table 7:** The data-driven model optimizes the efficiency prediction results of the hydrocyclone separator.

Type	Diameter of overflow outlet/mm	Depth of overflow tube /mm	Length of small cone/mm	Separation efficiency
**Before optimization**	10	15	80	89
**After optimization**	6	20	60	93

## Results validation

### Numerical simulation validation

**Fig 9:**
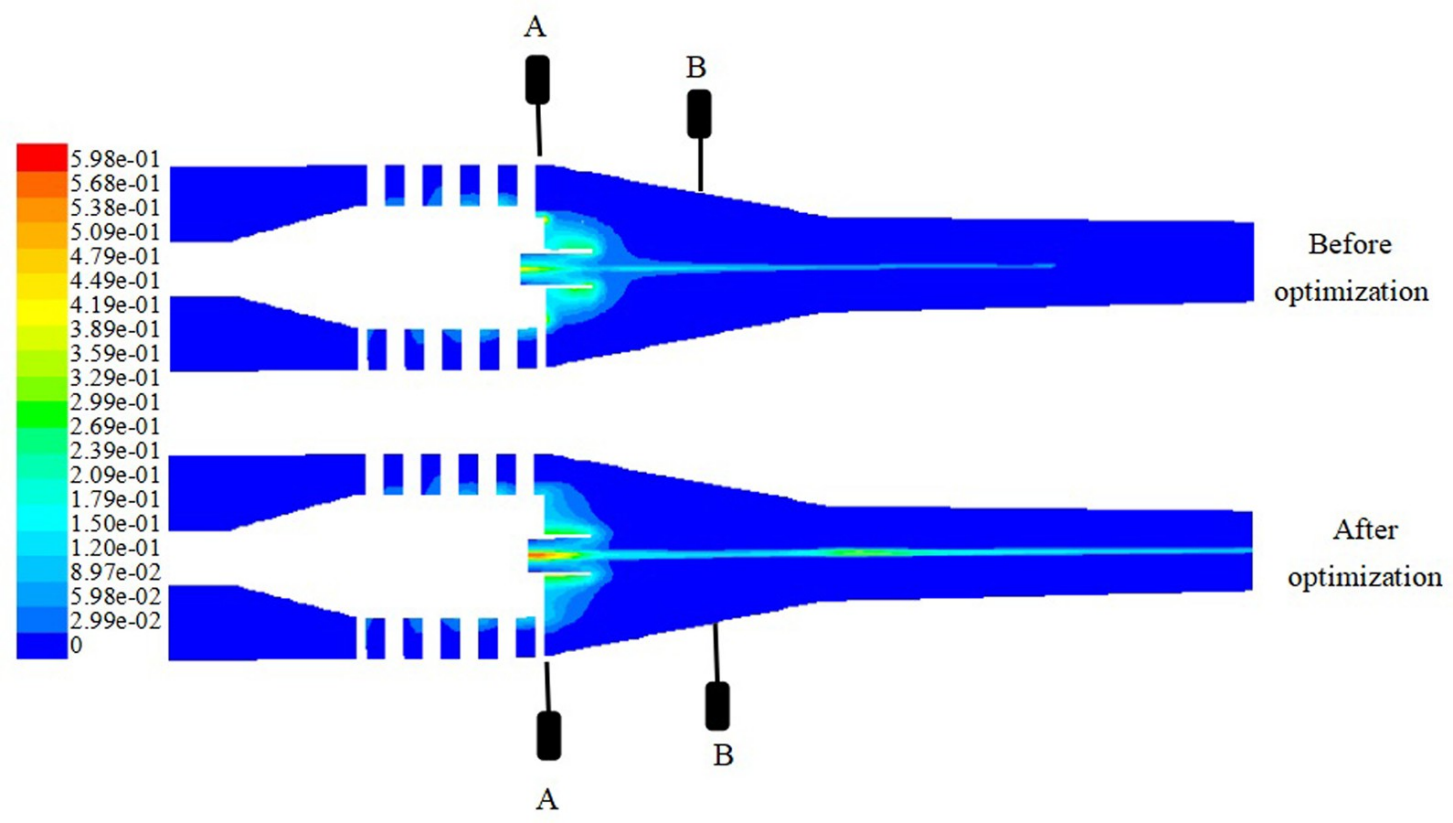
Oil phase volume fraction distribution cloud map.

Fig 9 shows the distribution of volume fraction of oil phase in the cross section under the structural parameters of two hydrocyclones before and after optimization by response surface methodology. From the distribution nephogram of hydrocyclone before and after optimization, it can be seen that the oil phase of the optimized hydrocyclone is gathered at the axial position and reaches the maximum volume fraction at the outlet of overflow pipe. The red area of oil phase in the nephogram is obviously different from that before optimization. The oil phase position of the optimized hydrocyclone model is closer to the axial position than that of the model before optimization, and the volume fraction of oil phase at the axial position is higher than that before optimization. Therefore, The optimized model has significantly improved the efficiency of crude oil separation.

**Fig 10:**
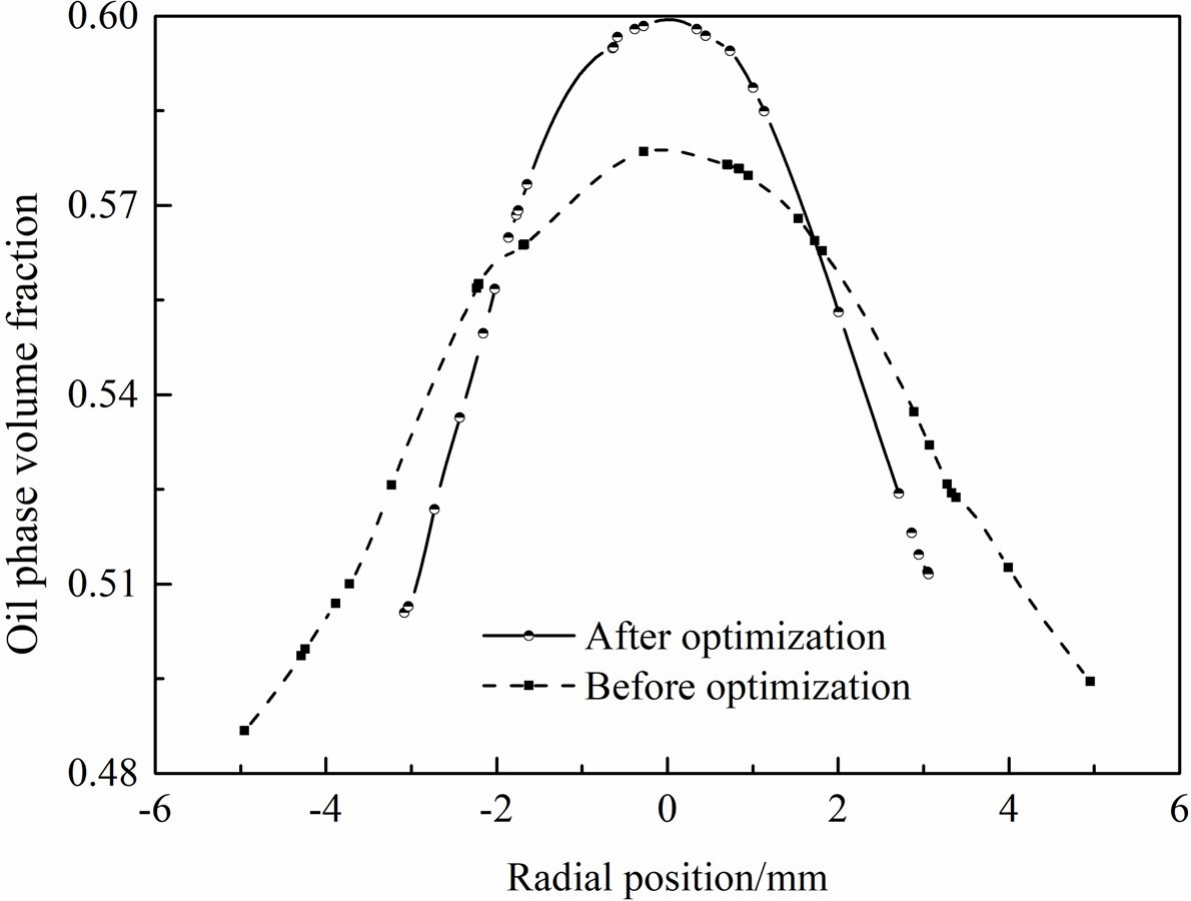
Oil phase volume fraction distribution curve.

Fig 10 shows the distribution curve of oil phase volume fraction at section A in both structures before and after optimization using response surface methodology. It can be seen from Fig 10 that the optimized axial guide cone hydrocyclone structure is 3% higher than the oil phase volume fraction at the shaft center before optimization, and the optimized model oil phase volume fraction curve is more symmetrical than before optimization. The trend of the curve verifies the accuracy of the results of the response surface optimization method.

**Fig 11:**
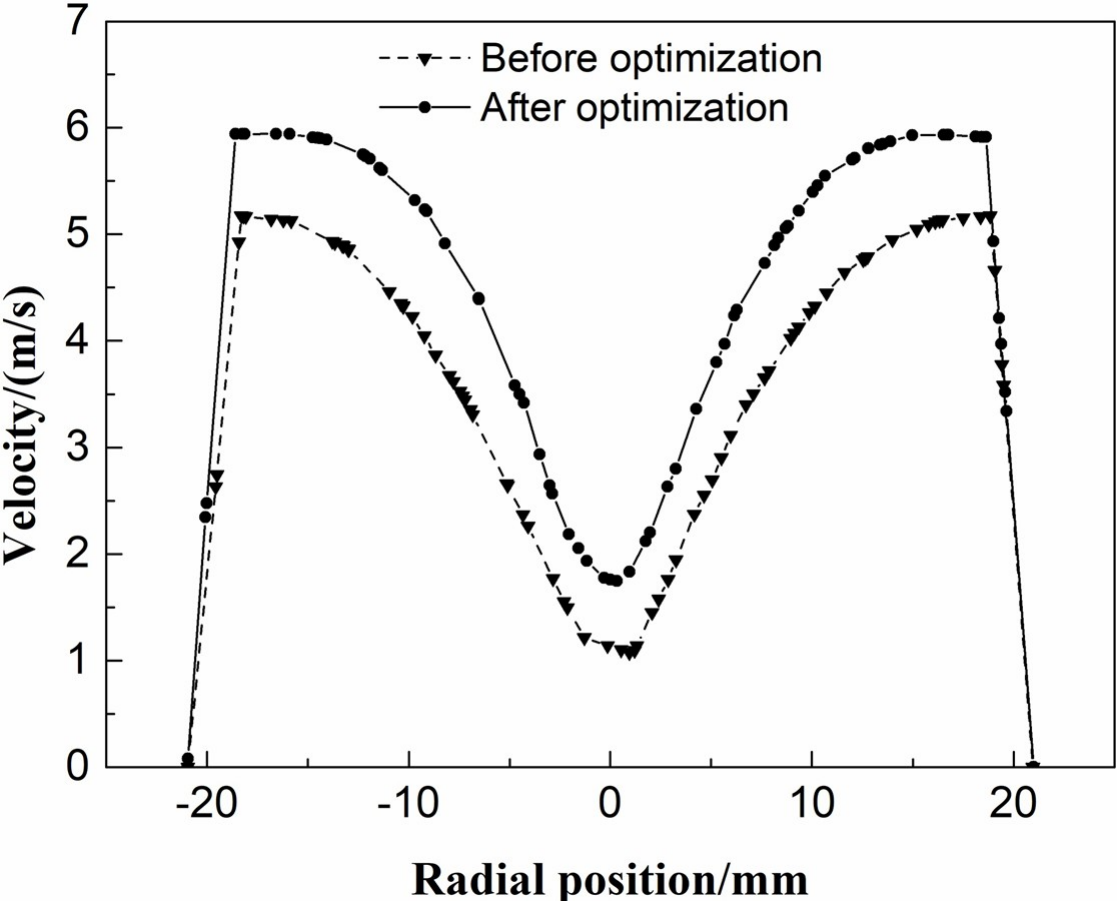
Velocity distribution curve.

Fig 11 shows the velocity comparison curves at the B-section position in the two structures before and after optimization using the response surface method. From Fig 11, it can be seen that the distribution curves before and after optimization have similar basic shapes, all of which are in an M-shape. The symmetry of the velocity distribution after optimization is better than that before optimization. After optimization, the velocity deviation at the center of the circle before optimization is eliminated, and it is not easy to form twisted secondary vortex. Moreover, the optimized speed and the difference in internal and external swirl velocity both increase, which is conducive to obtaining greater centrifugal force when the oil droplets move inside the cyclone separator, making it easier to be thrown towards the sidewall, thereby improving separation efficiency.

### Experimental validation

**Fig 12:**
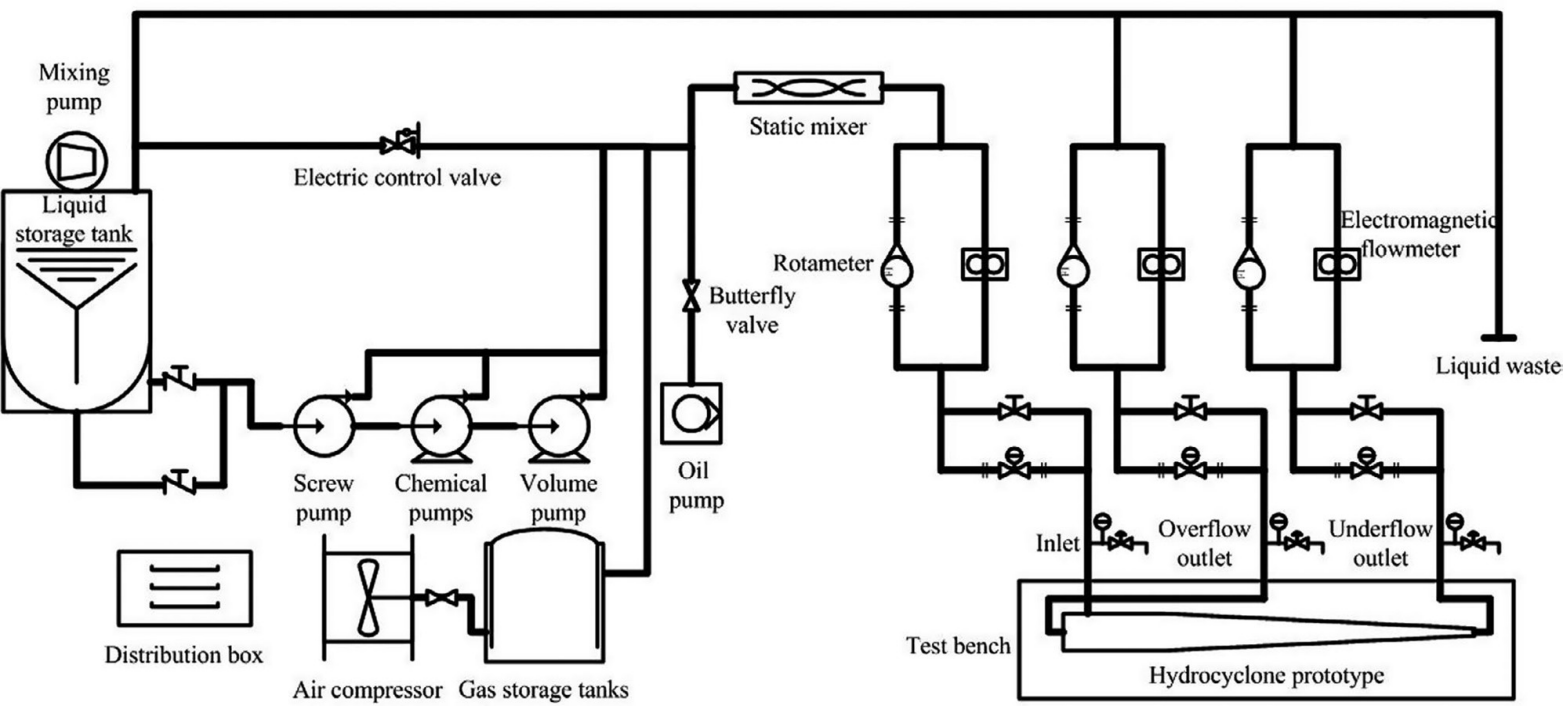
Laboratory test equipment.

The experimental prototype of two kinds of hydraulic cyclone separators before and after processing optimization was carried out and laboratory tests was carried out. The test process and device were shown in Fig 12. The oil phase enters the oil-water mixing tank through the plunger metering pump, and then enters the mouth tube through the screw pump after mixing. The working frequency of the screw pump is adjusted by the frequency converter to control the liquid intake. At the same time, the split ratio is controlled by adjusting the bottom flow and overflow outlet valves. The separation efficiency of the two test prototypes was adjusted when the shunt ratio varied in the range of 5% ∼ 35%.

**Fig 13:**
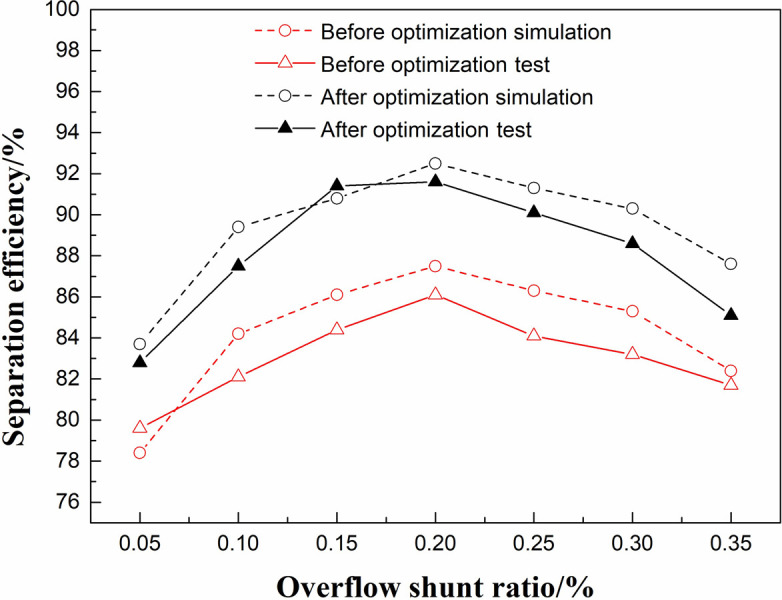
Separation efficiency comparison.

The simulation and experimental data before and after optimization were compared, as shown in Fig 13. It can be seen that the experimental results are in good agreement with the numerical simulation separation efficiency results, and the experimental results also prove that the separation efficiency of the optimized structural model is 3.4% higher than that of the initial model before optimization, which is a strong supplement to the results of the response surface optimization method.

## Conclusion

1. According to the experimental model P value, it is determined that the overflow tube diameter, the overflow tube insertion depth and the length of the cone section have significant effects on the separation efficiency of the hydrocyclone separator.
2. Based on the response surface optimization method, the mathematical driving model of the hydrocyclone separator in the global response range is obtained, and the optimal structural parameters in the global response range are obtained.
3. Through numerical simulation and indoor experimental results verification, it has been proven that the optimized hydrocyclone has significantly improved the oil phase volume distribution, acceleration section speed, and separation efficiency at the overflow port compared to the pre optimized hydrocyclone, and the flow field distribution is more symmetrical and superior.
4. The response surface optimization method can be used to quickly optimize the structure of a hydrocyclone separator, making it easier to design a hydrocyclone separator with the best structural parameters.

## References

[1] Jiang MZ, Chang SY, Dong KX. Research on working condition diagnosis method of downhole oil-water separation system with sucker-rod pump[J]. Advances in Mechanical Engineering, 2023, 15(4): 16878132231162186. 10.1177/16878132231162186

[2] Jiang MZ, Cheng TC, Dong KX, Liu JT, Zhang HY. An efficient downhole oil/water separation system with sucker-rod pump[J]. SPE Production and Operations, 2020, 35(3): 522–536. 10.3969/j.issn.1673-5005.2019.05.009

[3] Zeng XB, Zhao L, Zhao WG, Hou MW, Zhu FQ, Fan GM, et al. Experimental study on a Novel Axial Separator for Oil-Water Separation[J]. Industrial and Engineering Chemistry Research, 2021, 59(48):21177–21186. 10.1021/acs.iecr.0c03913

[4] Liu H, Gao Y, Pei XH, Zheng GX, Zheng LC. Progress and prospect of downhole cyclone oil-water separation with single-well injection-production technology[J]. Acta Petrolei Sinica, 2018, 39(4):463–471. 10.7623/syxb201804010

[5] Wang DM, Wang Y, Zhang LL, Liu JT, Zhang HY, He ZG, Zhang Y. Matching technology and application effect of injection-production in the same well[J]. Journal of China University of Petroleum(Edition of Natural Science), 2023, 47(2):64–72. 10.3969/j.issn.1673-5005.2023.02.007

[6] Zhao C, Sun H, Li Z. Structural optimization of downhole oil-water separator[J]. Journal of Petroleum Science and Engineering, 2017, 148: 115–126. 10.1016/j.petrol.2016.09.033

[7] Xing L, Li XY, Jiang MH, Zhao LX, Cai M. Structure optimization of micro-hydrocyclone for ultra-low inlet flow rate[J]. Journal of Mechanical Engineering, 2022, 58(23):251–261. 10.3901/JME.2022.23.251

[8] Liu PK, Wang H, Zhang YK, Yang XH, Li XY, Jiang LY, Li F. Separation of super clean coal with two-stage cyclones and related characteristics[J]. International Journal of Coal Preparation and utilization, 2021, 42(12):3682–3697. 10.1080/19392699.2021.1990893

[9] Xing L, Li JY, Zhao LX, Jiang MH, Han GX. Structural optimization of downhole hydrocyclones based on response surface methodology[J]. China Mechanical Engineering, 2021, 32(15):1818–1826. 10.3969/j.issn.1004-132X.2021.15.007

[10] Liu L, Zhao LX, Reifsnyder S, Gao S, Jiang MZ, Huang XQ. Analysis of hydrocyclone geometry via rapid optimization based on computational fluid dynamics[J]. Chemical Engineering and Technology, 2021, 44(9): 1693–1707. 10.1002/ceat.202100121

[11] Liu Y, Zhao LX, Zhang S, Liu L, Xu BR. Research and application of filtering -swirl coupling technology in heterogeneous separation[J]. Journal of Mechanical Engineering, 2022, 58(4):120–154. 10.3901/JME.2022.04.120

[12] Wei KF, Zhao Q, Kang CK, Zhang S, Cui BY, Liu KS. Effect of dual cone structure on the flow field characteristic and separation performance of hydrocyclones[J]. Metal Mine,2023(3):214–221.

[13] Liu HY, Han TL, Wang Y, Huang QS. Influence of new outlet configurations with baffle on hydrocycloneon separation performance[J]. CIESC Journal, 2018, 69(5): 2081–2088. 10.11949/j.issn.0438-1157.20171066

[14] Zhao LX, Zhong S, Zhao XF, Li G, Ma B. A structural simulation analysis of the neotype porous overflow-pipe hydrocyclone[J]. China Petroleum Machinery, 2012, 40(3):62–66.

[15] Jiang MH, Wang HJ, Xing L, Zhang Y. Study on separation performance of axial guide cone hydrocyclone[J]. China Petroleum Machinery, 2017, 45(10):86–91.

[16] Gao SL, Wei DZ, Han C, Hu RB. Influence of apex diameter and cone angle on the flow field inside hydrocyclone[J]. Journal of Northeastern Universit(Natural Science), 2010, 31(5):728–732.

[17] Xing L, Jiang MH, Zhang Y, Xiong F. Analysis of droplet coalescence and breakage characteristics in axis-in hydrocyclone with diversion-cone[J]. Journal of China University of Petroleum(Natural Science Edition), 2019, 43(2):140–147. 10.3969/j.issn.1673-5005.2019.02.017

[18] Zhang Y, Xing L, Zhang Y, Jiang MH. Effects of discrete phase entering positions on separation performance of a hydrocyclone[J]. Journal of Chemical Engineering of Chinese Universities, 2017, 31(6):1311–1317. 10.3969/j.issn.1003-9015.2017.06.008

[19] Rumsey CL, Rivers SM, Morrison JH. Study of CFD variation on transport configurations from the second drag-prediction workshop[J]. Computers and fluids, 2005, 34(7):785–816. 10.1016/j.compfluid.2004.07.003

[20] Wang J, Priestman GH, Tippetts JR. Modelling of strongly swirling flows in a complex geometry using unstructured meshes[J]. International Journal of Numerical Methods for Heat and Fluid Flow, 2006, 16(8): 910–926. 10.1108/09615530610702069

[21] Grady SA, Wesson GD, Abdullah M, et al. Prediction of 10-mm hydrocyclone separation efficiency using computational fluid dynamics[J]. Filtration & separation, 2003, 40(9): 41–46. 10.1016/S0015-1882(03)00930-3

[22] Gatski TB, Jongen T. Nonlinear eddy viscosity and algebraic stress models for solving complex turbulent flows[J]. Progress in Aerospace Sciences, 2000, 36(8): 655–682. 10.1016/S0376-0421(00)00012-9

[23] Cokljat D, Slack M, Vasquez SA, et al. Reynolds-stress model for Eulerian multiphase[J]. Progress in Computational Fluid Dynamics, An International Journal, 2006, 6(1–3): 168–178. 10.1504/PCFD.2006.009494

[24] Azimian M, Bart H J. Numerical analysis of hydroabrasion in a hydrocyclone[J]. Petroleum Science, 2016, 13: 304–319. 10.1007/s12182-016-0084-7

[25] Wang L, Zheng Z, Wu Y, et al. Numerical and experimental study on liquid-solid flow in a hydrocyclone[J]. Journal of Hydrodynamics, Ser. B, 2009, 21(3): 408–414. 10.1016/S1001-6058(08)60164-X

[26] Patra G, Velpuri B, Chakraborty S, et al. Performance evaluation of a hydrocyclone with a spiral rib for separation of particles[J]. Advanced Powder Technology, 2017, 28(12): 3222–3232. 10.1016/j.apt.2017.10.002

[27] Launder BE, Spalding DB. The numerical computation of turbulent flows. Computer Methods in Applied Mechanics and Engineering[J].1974,3:269–289. 10.1016/0045-7825(74)90029-2

[28] Zhou YY, Zhang QC, Wang H, Zhou P, Chai TY. Ekf-based enhanced performance controller design for nonlinear stochastic systems[J]. IEEE Transactions on Automatic Control, 2017, 63(4): 1155–1162. 10.1109/TAC.2017.2742661

[29] Ren MF, Zhang QC, Zhang JH. An introductory survey of probability density function control[J]. Systems Science and Control Engineering, 2019, 7(1): 158–170. 10.1080/21642583.2019.1588804

[30] Liu W, Wang SL, Dong KX, Cheng TC. Research on optimization of perforation parameters for formation fractures based on response surface optimization method[J]. Plos One, 2021, 16(8):e0255793. 10.1371/journal.pone.0255793 PMC8372901 34407089

[31] Li L, Zhang S, He Q, Hu XB. Application of response surface methodology in experiment design and optimization[J]. Research and Exploration in Laboratory, 2015; 34(8): 41–45.

[32] Choi JH, Kim DJ, Chun YD, Han PW, Koo DH, Lee J. Approximate optimization for minimum torque ripple of three phase switched reluctance motor using response surface modeling[J]. International Journal of Applied Electromagnetics and Mechanics, 2012, 39(14):825–833. 10.3233/JAE-2012-1548

[33] Šereš Z, Maravić N, Takači A, et al. Treatment of vegetable oil refinery wastewater using alumina ceramic membrane: optimization using response surface methodology[J]. Journal of Cleaner Production, 2016, 112: 3132–3137. 10.1016/j.jclepro.2015.10.070

[34] Mangili I, Lasagni M, Huang K, et al. Modeling and optimization of ultrasonic devulcanization using the response surface methodology based on central composite face-centered design[J]. Chemometrics and Intelligent Laboratory Systems, 2015, 144: 1–10. 10.1016/j.chemolab.2015.03.003

